# The Effect of the Back-Pressure Changes in an Exhaust System on Vibration When Attaching a Variable Device during Idling

**DOI:** 10.3390/s22113985

**Published:** 2022-05-24

**Authors:** Il-Seok Kang, Sung-Mo Yang

**Affiliations:** 1 Department of Mechanical System, Korea Polytechnic Colleges, Muan 58542, Korea; 2 Department of Mechanical System Engineering, Jeonbuk National University, Jeonju 54896, Korea; yangsm@jbnu.ac.kr

**Keywords:** exhaust system, back pressure, valve overlap, idling vibration, variable device

## Abstract

The vibration of the ignition frequency component of the engine during idling causes driver discomfort. To minimize this, an optimal exhaust system with a variable device that can exert optimal pressure is required. In this study, the geometry of the variable device was designed in orifice and cylinder types. Next, the designed variable devices were implemented in a conventional exhaust system with an X chamber. A comparative analysis was conducted to propose an optimal geometry through back-pressure and vibration measurements. During the experiment, the orifice geometry exhibited higher back pressure than the conventional geometry and a large difference in back pressure before and at the exhaust gas merging position. Furthermore, the orifice geometry showed a 2.56% increase in the vibration of the 1X component in the Y-axis direction. By contrast, the cylinder geometry exhibited slightly higher back pressure than the conventional geometry and the smallest difference in back pressure before and at the exhaust gas merging position. The cylinder geometry showed a 2.45% reduction in the vibration of the 1X component in the Y-axis direction.

## 1. Introduction

The exhaust system is the main component used in vehicles to reduce the toxic exhaust gas, noise, and vibration generated from the engine. In various vehicles mass-produced in recent years, the exhaust system is appropriately tuned to improve the exhaust sound and performance of the engine, according to their characteristics. The owners of older vehicles also attempt to tune their exhaust systems to arouse emotions and express their personality.

In the exhaust system, various elements, such as the manifold, catalyst, resonator, and muffler, are connected to the exhaust pipe. During the tuning of the exhaust system, the outer diameter of the pipe is increased, or its geometry is changed naturally, while each element is modified according to tastes and purposes.

For tuning, however, the pressure of the exhaust gas that flows in the exhaust system should be considered. This is because the back-pressure change caused by modifying the exhaust pipe size and geometry of the exhaust system affects the power, exhaust emissions, noise, and vibration of the engine [1,2,3].

In general, the noise and vibration that are generated from the engine are reduced through several mounts, which is particularly effective in the high-frequency range, but still causes unpleasant vibrations to the driver in the low-frequency region [4,5].

Representative noise types that may typically occur in vehicles include noise radiating from the engine, exhaust noise from the muffler, and the booming noise that is transmitted to the interior through the vehicle body. Among them, the booming noise is the main cause of discomfort in people. The booming noise is generally classified into idling booming from the ignition frequency component of the engine, the booming of the power transmission system, including the transmission and the drive shaft, the booming of the vehicle body caused by resonance, and the booming noise caused by the transmission of exhaust and intake noises [6,7,8].

The main objective of this study is to reduce the vibration of the ignition frequency component of the engine during idling. This is because vibration is the main feature that is used to evaluate the comfort of vehicles during idling [9], and since unpleasant vibrations can be experienced for long periods along the feet and lumbar spine when vehicle users are in a sitting position, reducing the vibration during idling is very important to minimize this [10,11,12]. 

ISO 2631-1 defines the frequency range that is transmitted to the human body when it is in a sitting position and affects human health as 0.5 to 80 Hz [13]. In particular, the 1~40 Hz frequency range, the frequency range is the ignition frequency range that is transmitted from automobile engines, causes whole-body vibration.

Therefore, it is necessary to minimize the exposure to vibrations corresponding to the engine ignition frequency range. The engine used in this study was a naturally aspirated V6 engine, which involves three explosions per revolution. Owing to the vertically arranged engine characteristics, the vibration (lurching) of the engine from side to side is transmitted to the vehicle body and causes driver discomfort at low RPMs, such as idling. During idling, in particular, the flame speed is reduced because of the backflow of the exhaust gas caused by the valve overlap, which may cause abnormal combustion. This causes vibration, which may increase driver discomfort along with irregular explosions of the engine [14,15,16,17].

In addition to the exhaust-gas backflow, various phenomena, such as inadequate fuel, incorrect ignition timing, carbon deposits in the combustion chamber, and poor fuel injection, can cause abnormal combustion. To address these, many studies have been conducted [18,19,20,21]. 

Therefore, an exhaust system that can minimize the exhaust-gas backflow during idling and maximize the power performance of vehicle engines with secured flow areas during high-speed driving is required. In this study, a dual exhaust system with an X-shaped confluence chamber was adopted. This exhaust system can maximize the power of the engine during high-speed driving by securing the flow area, and it can reduce exhaust emissions and vibration by exerting stable back pressure at low speeds.

However, the vibration generated from the engine during idling is the main cause of driver discomfort. To minimize this discomfort, stable combustion should be maintained by minimizing the backflow of the exhaust gas that flows in the exhaust system. To this end, the flow area needs to be properly reduced during idling, and a variable device designed with a geometry that can minimize flow resistance should be separately installed at the same time. In addition, the exhaust-gas pressure change should be minimized during the application of the variable device, and the flow velocity should be high at the position where the variable device is applied.

In general, a flap variable device of the butterfly-valve type is installed in exhaust systems, in the exhaust pipes of recently launched diesel vehicles equipped with turbochargers. The device is installed to adjust the back pressure discharged from the engine at low speeds. In the case of naturally aspirated gasoline engines, however, a flap-type variable device is installed in a separate exhaust pipe, as it affects the back pressure at low speeds. It is opened during high-speed driving, or it is used to generate rough exhaust sound [22].

Therefore, in this study, a variable device with a geometry that can optimize the back pressure of the exhaust system during idling was devised based on a naturally aspirated gasoline engine. In addition, the back-pressure change in the exhaust system and the vibration of the vehicle body were measured to determine the optimal geometry.

## 2. Prototype Design

In this study, the orifice and cylinder types were selected as the basic geometry of the variable device, and their detailed sizes were determined to reduce the flow area of the conventional exhaust pipe by approximately 15%. 

**Figure 1:**
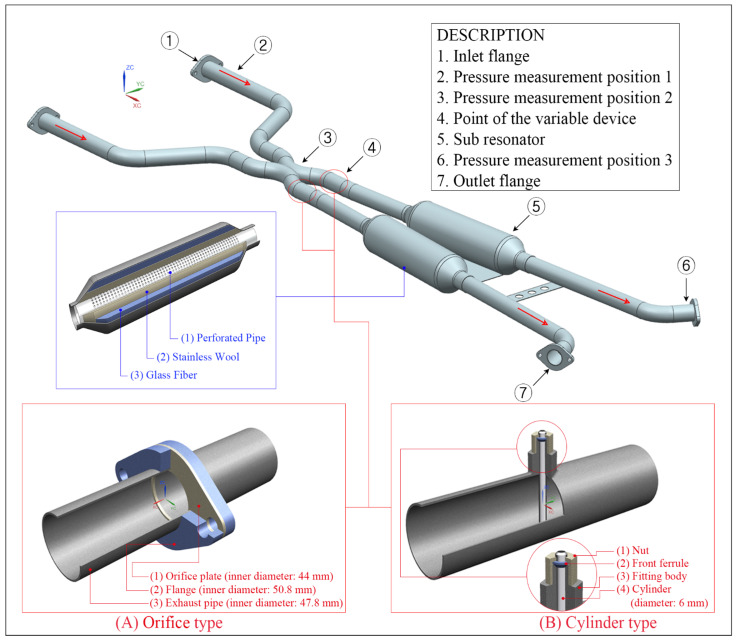
Description and schematic diagram of the designed exhaust system and variable device. (**A**) Designed orifice valve type; (**B**) Designed cylinder type.

Figure 1 shows the design of the prototype exhaust system and variable devices used in the experiment. Descriptions of the designed exhaust system and each position are provided in the figure, and the structures of the variable devices and sub-resonators used in the exhaust system can be seen. 

Figure 1A,B show the orifice and cylinder valve types, respectively. These devices were designed under the assumption of a closed state. Subsequently, the geometry that showed the best experiment results was combined with an electric motor or an air cylinder to design the driving part of the valve. The variable devices were designed to be installed at position 4, which was approximately 965 mm away from the X-axis origin of the coordinate system shown in the figure.

## 3. Experiment

**Figure 2:**
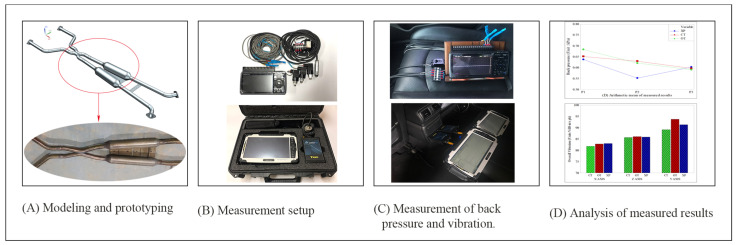
Schematic diagram of the experimental procedure. (**A**) Exhaust system design and prototyping; (**B**) back-pressure and vibration measurement settings; (**C**) back-pressure and vibration measurement; (**D**) analysis of measured results.

The vehicle used in the experiment of this study was a company I sports sedan equipped with a V6 naturally aspirated engine. Given that this is a rear-wheel-drive vehicle, the exhaust system shown in Figure 2A was installed. The exhaust system used was designed, as shown in Figure 1, to maintain the exhaust gas discharged from the engine in a stable-back-pressure state, and Table 1 shows the engine specifications of the vehicle. The back pressure of the exhaust system and the vibration of the vehicle body were measured to analyze the influence of the vibration generated from the engine.

For the experiment, measurements were performed in a conventional exhaust system without a variable device, with the orifice-valve-type variable device, and with the cylinder-type variable device. Figure 2 shows the experimental procedure of this study. Figure 2A shows the design and prototyping stage, and Figure 2B shows the measurement setting stage. Figure 2C shows the back-pressure and vibration measurement stage, and Figure 2D shows the analysis of the measurement results.

### 3.1. Back-Pressure Measurement

**Figure 3:**
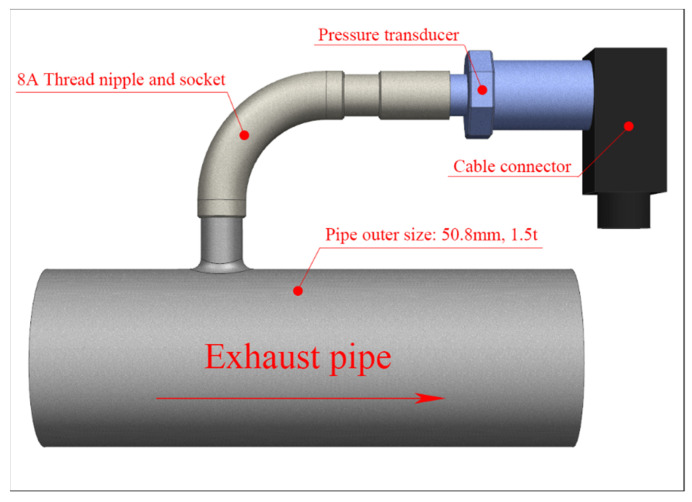
Schematic diagram of the thread socket and pressure transducer connected to the exhaust pipe.

A pressure transducer with the specifications listed in Table 2 was used to measure the back pressure in the exhaust system, and a data logger with the specifications listed in Table 3 was used to collect the measured data. The pressure transducer was installed at each measurement position using an 8A thread nipple, as shown in Figure 3. Based on the X-axis origin of the coordinate system shown in Figure 1, the pressure transducer was installed at distances of 100 mm (pressure measurement position (1), 800 mm (pressure measurement position (2), and 2400 mm (pressure measurement position (3) [1,2]. At the time of measurement, the air temperature was approximately 10 °C, and the humidity was approximately 50%. The temperature of the exhaust gas from the engine was measured to be approximately 120 °C at pressure measurement position 1. The back pressure was measured three times for 3 min during idling, and the engine RPM was found to be 680 ± 20 during the measurement.

### 3.2. Vibration Measurement

**Figure 4:**
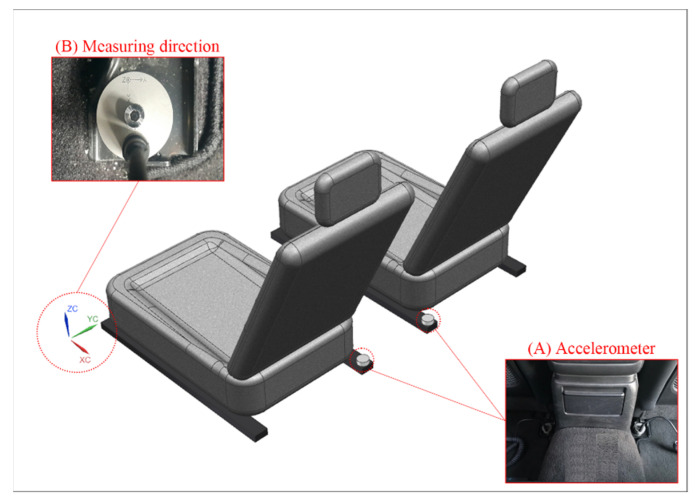
Accelerometer installed to measure the vibration. (**A**) Accelerometer installed on a seat frame; (**B**) measurement direction of the accelerometer.

Table 4 shows the specifications of the equipment used to measure the precise vibration of the vehicle body. The acceleration sensor used for vibration measurement was of the piezoelectric type, and ICP Triaxial Accelerometer manufactured by IMI was used. ISO 2631-1 was referred to for vibration measurement, and accelerometers were installed in the driver’s seat and the passenger seat, as shown in Figure 4A. Figure 4B shows the measuring direction. The Z-axis represents the vehicle body direction, X-axis represents the engine direction, and Y-axis represents the door direction [23,24,25,26]. Vibration is insufficient to quantify physical effects on the human body. Therefore, the vibration velocity level is expressed as VdB, and the VdB is a logarithmic scaling of vibration magnitude. 

The vibration velocity level in dB is abbreviated VdB, and VdB is defined as Equation (1), where “Lv” is the velocity level in decibels, “v” is the RMS velocity amplitude, and “$v_{ref}$” is the reference velocity amplitude.


(1) $$Lv=20\times log_{10}\left(\frac{v}{v_{ref}}\right)$$

During the measurement, the 0–200 Hz range was set as the low-range section. Ten measurements were performed during idling, and the components were analyzed.

## 4. Results and Discussion

### 4.1. Analysis of the Measured Back Pressure

**Figure 5:**
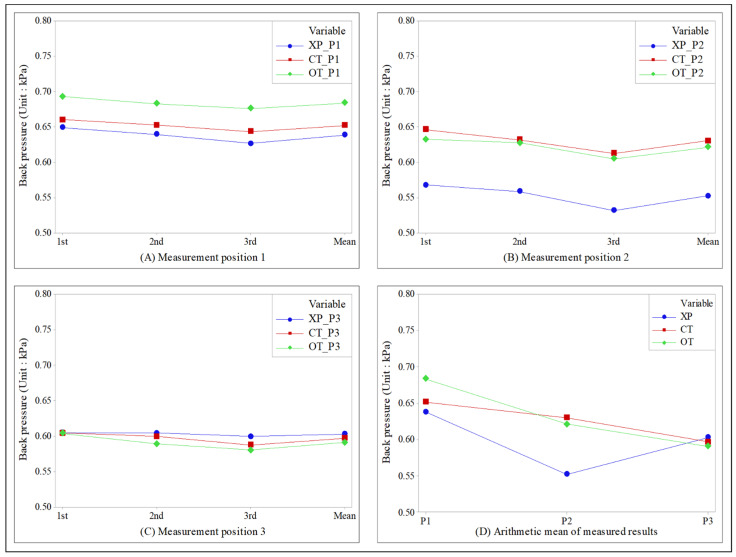
Time-series plot of the back pressure measured at each measurement position (**A**) at measurement position 1; (**B**) at measurement position 2; (**C**) at measurement position 3; (**D**) at each measurement position. XP: X pipe; OT: orifice type; CT: cylinder type.

The exhaust-gas pressure measurement results are shown in Figure 5 using time-series plots. 

Figure 5A shows the results measured at pressure measurement position 1. It can be seen that the orifice and cylinder types exhibited higher pressure than the conventional X pipe (XP) type. Based on the average value of the three measurements, the XP type showed 0.638 kPa. 

The orifice and cylinder types showed 0.684 kPa and 0.652 kPa, respectively, which were 7.21% and 2.19% higher than the conventional type. These results confirm an increase in back pressure after the application of a variable device. 

In particular, an increase in back pressure at measurement position 1 may lead to an increase in the backflow of burnt exhaust gas during the valve overlap period, thereby causing abnormal combustion. Therefore, the cylinder type that minimizes the back pressure increase was expected to exhibit higher performance than the orifice type.

Figure 5B shows the measurement results obtained at pressure measurement position 2. The orifice and cylinder types exhibited higher pressure than the conventional XP type. Based on the average value of the three measurements, the XP type showed 0.552 kPa. The orifice and cylinder types showed 0.621 kPa and 0.630 kPa, respectively, which were 12.5% and 14.13% higher than the conventional type. These results also confirm that the back pressure increased after the application of a variable device. The important issue to be examined, however, is the pressure difference between measurement positions 1 and 2. A significant pressure difference means a significant change in the flow velocity. In particular, as the X chamber, which was expected to merge the exhaust gas, reduced the cross-sectional area of the flow to increase the flow velocity while stabilizing the exhaust gas pressure, the back pressure at measurement position 2 showed the lowest value. Therefore, the flow velocity should be stabilized by minimizing the difference in back pressure between measurement positions 1 and 2. For the cylinder type, the pressure increased by 14.13%, as compared to the geometry of the conventional XP type, but the pressure difference compared with measurement position 1 was found to be 3.37%.

For the orifice geometry, the pressure increased by 12.5% compared to the conventional geometry, but the pressure difference compared with measurement position 1 was 9.21%. These results confirm that the back-pressure formation of the cylinder type is more stable than that of the orifice type. At measurement position 2, the conventional XP geometry showed the lowest pressure. For the exhaust system with a variable device, however, the back pressure increases owing to the flow resistance generated at the position where the orifice or cylinder variable device was applied. The pressure was the lowest at the position where the variable device was installed.

Figure 5C shows the measurement results obtained at pressure measurement position 3. The orifice and cylinder types exhibited slightly lower results than the conventional XP type. Based on the average value of the three measurements, the XP type showed 0.603 kPa. The orifice and cylinder types showed 0.591 kPa and 0.597 kPa, respectively, which were 2.03% and 1.0% lower than the conventional type.

Figure 5D shows the arithmetic mean of the measured values at each measurement position for each type. The orifice type exhibited the highest pressure at measurement position 1, whereas the cylinder type exhibited the highest value at measurement position 2. At measurement point 3, the conventional XP type showed the highest value. By contrast, the lowest pressure was generated by the XP type at measurement positions 1 and 2 and by the orifice type at measurement position 3. In addition, the back pressure increased at measurement position 1, and the pressure difference measured at positions 1 and 2 was intuitively compared.

### 4.2. Analysis of the Measured Vibration

In the vibration measurement analysis, the overall vibration was analyzed using a bar plot, and the ignition frequency components were analyzed using scatterplots based on the results obtained in three axial directions. 

**Figure 6:**
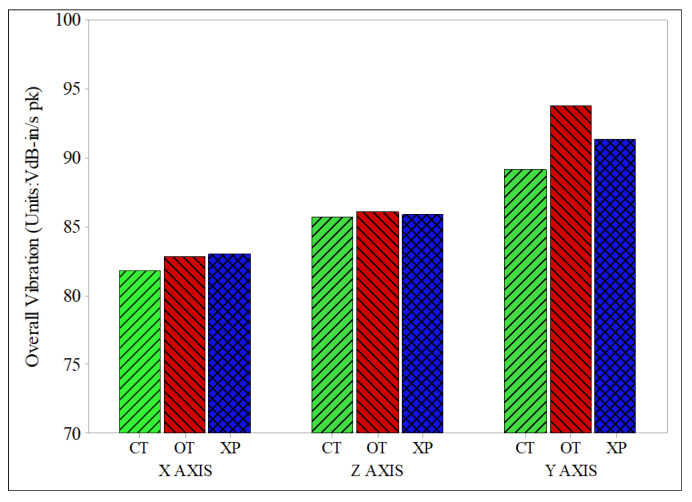
Results of the overall vibration measured in the X-, Z-, and Y-axis directions.

Figure 6 shows the arithmetic mean values of the overall vibration obtained through ten measurements in the passenger seat and the driver’s seat, respectively, in a bar plot. The largest vibration was clearly observed in the Y-axis direction and the smallest vibration in the X-axis direction. In the Y-axis direction, which exhibited the largest vibration, the exhaust system of the conventional XP type showed 91.445 VdB, and that of the orifice type showed 93.790 VdB (2.56% higher). The cylinder-type system showed 89.200 VdB (2.45% lower), which was 5.14% lower than that of the orifice-type system. These results were analyzed through a comparison with the back-pressure measurement. The orifice type showed 7.21% higher pressure than the conventional XP type at pressure measurement position 1, and the largest pressure difference, of 9.21%, between measurement positions 1 and 2. Therefore, it appears that the orifice type exhibits relatively larger vibration than the XP type, because abnormal combustion increases under the influence of back pressure. The cylinder type showed only a 2.19% higher back pressure than the conventional XP type, and the smallest pressure difference, of 3.37%, between measurement positions 1 and 2. Therefore, it appears that the vibration is lower, compared to the XP type, because abnormal combustion is minimized under the influence of stable back pressure. 

**Figure 7:**
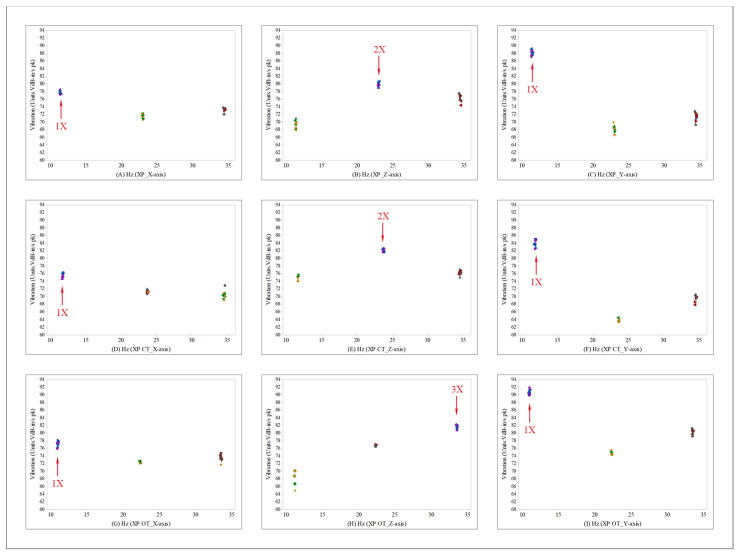
Scatterplot of the ignition frequency and vibration values measured from the driver’s seat.

**Figure 8:**
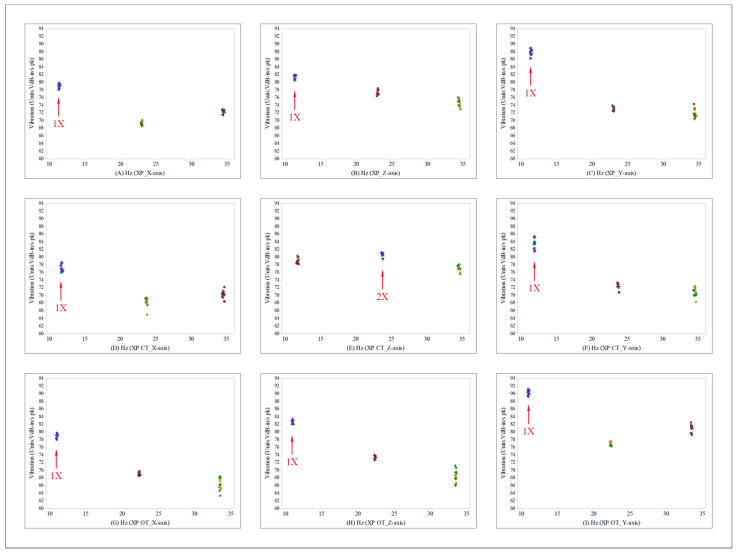
Scatterplot of the ignition frequency and vibration values measured in the passenger seat.

Figure 7 and Figure 8 show the measured ignition frequency components and vibration values in scatterplots. Based on an engine RPM of 680 ±20 during idling, the 1X component of the ignition frequency was found to be 11.33 Hz, and the 2X component was 22.66 Hz. The 3X component was 34 Hz. Figure 7 shows the results measured in the driver’s seat in scatterplots. In the figure, the XP type is shown in (A–C), and the cylinder type is shown in (D–F). The orifice type is shown in (G–I). The vibration in the X-axis direction is shown in (A,D,G); the 1X component exhibited the largest vibration. The vibration in the Z-axis direction is shown in (B,E,H). The 2X component exhibited the largest vibration in (B,E), whereas the 3X component exhibited the largest vibration in (H). The vibration in the Y-axis direction is shown in (C,F,I); the 1X component exhibited the largest vibration. Therefore, the comparison with the overall vibration shown in Figure 6 confirms that the largest vibration occurred in the Y-axis and that the 1X component was the main vibration source. It was also confirmed that the scatterplot in the Y-axis decreased for the cylinder type, as compared to the conventional XP type, and that the scatterplot in the Y-axis increased for the orifice type.

Figure 8 shows the results measured in the passenger seat in scatterplots. In the figure, the XP type is shown in (A–C), and the cylinder type is shown in (D–F). The orifice type is shown in (G–I). The vibration in the X-axis direction is shown in (A,D,G); the 1X component exhibited the largest vibration. The vibration in the Z-axis direction is shown in (B,E,H). The 1X component exhibited the largest vibration in (B,H), whereas the 2X component exhibited the largest vibration in (E). The vibration in the Y-axis direction is shown in (C,F,I); the 1X component showed the largest vibration. Therefore, in Figure 8, the largest vibration also occurred on the Y-axis, and the 1X component was the main vibration source. The scatterplot in the Y-axis decreased for the cylinder type, compared to the conventional XP type, whereas the scatterplot in the Y-axis increased for the orifice type.

Therefore, the comparison with the back pressure results mentioned previously shows that the back pressure was the highest at measurement position 1. Furthermore, the orifice type, which exhibited a large pressure difference between measurement positions 1 and 2, increased the vibration of the 1X component in the Y-axis direction because of the increase in the abnormal combustion. The cylinder type exhibited a relatively small back pressure at measurement position 1 and a small pressure difference between measurement positions 1 and 2. Therefore, the cylinder type reduced the vibration of the 1X component in the Y-axis direction because of the reduction in the abnormal combustion.

## 5. Conclusions

In this study, orifice-type and cylinder-type variable devices were implemented in an exhaust system. Next, the back pressure of the exhaust system and the vibration of the vehicle body were measured to analyze the effect of the back pressure formed in the exhaust pipe on the vibration of the vehicle body during idling. The analysis results of this study are as follows.

According to the measurement results of back-pressure under the application of the various devices, the orifice type showed the highest pressure at measurement position 1, which was 7.21% higher than the conventional XP type, and a pressure difference of 9.21% between measurement positions 1 and 2. It exhibited the largest overall vibration, of 93.790 VdB, in the Y-axis direction, which was 2.56% higher than the conventional XP type. The main vibration component was the 1X component in the Y-axis direction, which corresponded to an ignition frequency of 11.33 Hz.

The cylinder type showed a pressure that was only 2.19% higher than that of the conventional XP type at measurement position 1 and a pressure difference of 3.37% between measurement positions 1 and 2. It exhibited the smallest overall vibration, of 89.200 VdB, in the Y-axis direction, which was 2.45% lower than the conventional XP type. The main vibration component was the 1X component in the Y-axis direction, which corresponded to an ignition frequency of 11.33 Hz.

Through the experimental results, it was confirmed that the largest vibration occurred at 11.3 Hz, which was the ignition frequency propagated from the engine. Vibrations of 1–40 Hz cause significant whole-body vibration; 11 Hz is the point at which headaches and abdominal pain can occur. Therefore, it was confirmed that the application of the cylinder-type variable device minimized the increase in pressure at measurement position 1, and as a result of minimizing the pressure difference between measurement positions 1 and 2, it was confirmed that the vibration of the ignition frequency was reduced. Compared with the results of previous studies [2], the application of the cylinder-type variable device is expected to exert stable back pressure and, thus, reduce exhaust emissions.

## Figures and Tables

**Table 1:** Specifications of the V6 engine of the vehicle used in the experiment.

Description	Specification
Type	V6, DOHC 24 valve
Bore and stroke size	95.5 × 86.0 mm (3696 cc)
Compression ratio	11:1
Maximum power and torque	330 hp/7000 rpm
	36.8 kgf∙m/5200 rpm

**Table 2:** Specifications of the pressure transducer used for back-pressure measurement.

Description	Specification
Type	Piezo-resistant
Measurable Range	−30~30 kPa
Accuracy	±0.25%
Operating temperature range	−20~100 °C
Output type	4–20 mA (two wires)

**Table 3:** Specifications of the data logger used for data collection.

Description	Specification
Number of input channels	20 channels
Accuracy	Voltage ± 0.1%
Sampling interval	50 ms
Operating environment	0–45 °C, 5–85% RH

**Table 4:** Specifications of the vibration data collector used for the measurement.

Description	Specification
Measurements	Acceleration Velocity (by integration) Displacement
Sensor type	3-axis acceleration
Band width	0.5–40 kHz
Dynamic range	104 dB
Sampling rate	102.4 kHz

## Data Availability

Not applicable.
